# Morphometric analysis of the abducens nerve in the petroclival region

**DOI:** 10.3389/fsurg.2025.1574047

**Published:** 2025-04-24

**Authors:** M. Bach, M. J. Schmeisser, S. Schumann

**Affiliations:** ^1^ Institute of Anatomy, University Medical Center of the Johannes Gutenberg-University Mainz, Mainz, Germany; ^2^ Focus Program Translational Neurosciences, University Medical Center of the Johannes Gutenberg-University, Mainz, Germany; ^3^ Institute of Anatomy, Brandenburg Medical School, Neuruppin, Germany

**Keywords:** cranial nerve, dura mater, skull base, anatomical variation, neurosurgery

## Abstract

**Background:**

The abducens nerve (AN), our sixth cranial nerve, is responsible for the innervation of the lateral rectus muscle of the eye. The abducens nerve is a vulnerable structure at the skull base with its long intracranial course and complex topographic relationships. The AN anatomy in the petroclival region, where the nerve passes from the posterior to the middle cranial fossa, is of great interest for neurosurgical procedures. Despite detailed studies of its anatomy from the past 150 years, there is a need for more recent data on macroscopical and microscopical aspects of the AN in well defined populations.

**Methods:**

We investigated macroscopical variations and the number of nerve fibers of the AN in the petroclival region in German body donors.

**Results:**

In our histological samples (*n* = 24) we counted 4688 (+/−1,041) nerve fibers per AN. There was no correlation between sex, age and body side regarding the number of nerve fibers. In our macroscopic examination (*n* = 76 skull base sides), we found six duplications (four left-sided, two right-sided; 7.9%) and one triplication (right-sided; 1.3%) of the AN in the petroclival region. The AN triplication was further examined: Three nerve bundles pierce the dura mater separately and united before passing under the petrosphenoidal ligament (of Gruber).

**Conclusion:**

Variations of the AN in the petroclival region are not a rare phenomenon but occur very frequently. Consequently, we have developed a new classification system for AN variations. This knowledge might help neurosurgeons, as it prepares them to be aware of such variations and adapt their surgical approaches accordingly.

## 1 Introduction

Abducens nerve (AN) palsy is a disease of the sixth cranial nerve that causes the inability to contract the lateral rectus muscle (LRM) due to insufficient innervation. Consequently, the other extraocular muscles become more dominant, resulting in a lack of gaze control. Patients report diplopia and often compensate by rotating their heads to the affected side ( 1 ).

AN palsy is the most prevalent cranial nerve palsy in neurological emergency rooms with incidences between 4.66 and 11.3/100.000 inhabitants (2, 3). The dissection of the internal carotid artery in the cavernous sinus is the most common pathology leading to AN palsy due to the artery's close topographic relationship to the AN. Additionally, skull base fractures involving trauma settings, in particular the petrosal bone fractures (both longitudinal and transverse), can affect the AN in its course which then contributes to neurologic deficits (4, 5). Furthermore, systemic diseases, such as chronic hyperglycemia and chronic, degenerative neurological diseases like multiple sclerosis, have been shown to correlate to the development of AN palsy (3). Petroclival meningiomas and other tumors of the skull base, such as the facial schwannoma can infiltrate the structural integrity of the AN and its course. The AN and its surrounding structures, like the posterior clinoid process, are important landmarks for surgical orientation (6). For example, the AN's dural entrance at the clivus is one of the landmarks for the inferomedial paraclival triangle, which serves as a roadmap for surgical interventions at the skull base (7). The trans-petrosal approach of petroclival tumors (meningiomas or facial schwannomas) holds the risk of cranial nerve affection in this region (8). The AN is particularly vulnerable during those procedures because of its exposed position in both the posterior and middle cranial fossa and shows statistically significant postoperative complications (9). In order to perform these surgical interventions safely, it is crucial to know about the macroscopic anatomy and its topographic relations and possible variations, in particular the localizations of aberrant nerve branches, very well. The differences in the prevalence of occurrence among different ethnicities should additionally be considered.

In its ordinary course, the AN exits the brainstem at the pontomedullary junction and travels in its subarachnoid course through the prepontine cistern, piercing the dura at the clivus region. From the dural porus, meningeal layers accompany the nerve and form a pocket filled with cerebrospinal fluid around the nerve (10–12). The petroclival region is of particular interest regarding the AN vulnerability as the meningeal sheet of the AN is attached to the periosteal dura (13) and the moveability of the AN is restricted by fixations at the dural entrance (14) and the petrosphenoidal ligament (15). The nerve is running between the clival (inner) and periosteal (outer) dural layer in a meningeal sheet. These two layers are separated by the so-called petroclival venous confluence by Destrieux et al. (13). Ozveren et al. (16) revealed that the dural layer extends after the porus, following the nerve as it travels cranially up to the Dorello's canal. The arachnoid layer also extends in a similar manner. Joo et al. (17) found similar dural relations regarding the AN with the dural sleeve ending at the LRM.The AN usually travels below the petrosphenoidal ligament of Gruber in Dorello's canal (18, 19) and continues its course through the cavernous sinus before entering the orbit. The petrosphenoidal ligament of Gruber can be partly ossified, the so-called Foramen sphenopetrosum osseum anomalum (20).

Given the lack of recent studies on AN anatomy in a German population (both in terms of macroscopical variations and microscopical aspects), we investigated the number of AN fibers and its dural entrance. This information could be interesting for future surgical procedures at the skull base.

## 2 Materials and methods

This study was performed on adult human skull bases from 12 formalin-fixed body donors from the dissection course (five males and seven females) and 52 skull base specimens (26 right and 26 left sides) from the anatomical collection of the Institute of Anatomy, University Medical Center of the Johannes Gutenberg-University Mainz. The age of the 12 donors ranged between 65 and 95 years (mean age: 83.36). Skull base specimens were of unknown sex, age, and medical history. All body donors donated their bodies willingly for research and education. This study was approved by the ethical committee of Rhineland-Palatinate (2023-17245).

### 2.1 Histological examination

Semithin sectioning was used for the histological analysis. The tissue samples from the subarachnoid part of the abducens nerve were taken after opening the skull and removing the brain. All samples were taken from the last 0.5 cm of the AN before the dural porus. After fixation in glutaraldehyde, samples were placed in a phosphate buffer (3 × 15 min). Fixation was continued with 2% osmium tetroxide for two hours, followed by phosphate buffer. Dehydration was carried out with an ethanol series in ascending ethanol concentrations (50%–70%–90%–100%). The samples were then treated with a mixture of propylene oxide (PO) and epoxide resin (EPON). The propylene oxide was used as a transition solvent in this process. After three PO/EPON mixtures (1 h each) with an ascending portion of the epoxide resin, samples were placed in pure epoxy resin overnight. After pouring, polymerization followed at 65°C in an incubator for 2 days. Using the Metvacut E microtome (Reichert-Jung, Germany), the blocks were cut into 1 µm thick slices. Slices were stained with 1% azure II and 1% methylene blue (21). After the staining process was finished, samples were mounted using Cytoseal xyl.

The histological sections were imaged with a Leica MS 5 tripod (Leica Microsystems, Germany) and a JVC KY-F75U C mount digital camera (JVC, Yokohama, Japan) and digitally processed with the histology software Diskus (Version 4.807713).

### 2.2 Analyzed area

Using the Diskus software described above, an area of 225 × 175 µm^2^ was randomly selected over which a grid was placed. The grid consisted of 9 × 7 square boxes of 25 × 25 µm^2^ each. The total area of the mapped nerve cross-section was calculated using the software by outlining the nerve cross-section. A total of three different histological sections were evaluated for each nerve. An average value for the number of fibers per grid area and its total area was calculated from the three values per nerve. In addition, the simple standard deviation was determined in each case.

### 2.3 Counting method and calculations

The number of nerve fibers was counted manually using a mechanical hand counter. The “Forbidden Rule of Gunderson” method was used for manual counting. Nerve fibers on the square's left and bottom lines were not counted. However, those on an upper or right line are included (22). The number of nerve fibers of a grid box (225 × 175 µm^2^) was extrapolated to the previously determined total area of the nerve. Our calculations neglected the tissue shrinkage due to histological processing, as previous studies showed no significant difference when this factor was taken into account (23).

### 2.4 Statistical analysis

The data acquired by the study were expressed as mean values with standard deviation (+/−SD). *T*-test was used as a statistical tool. A *p*-value of <0.05 was considered to be statistically significant.

### 2.5 Macroscopical examination

52 skull base sides (26 right, 26 left) were examined macroscopically. The course of the AN was assessed by further anatomical dissection. The dural layer was carefully removed, so the course of the AN could be seen before passing under Gruber's ligament. Further investigation of its course in the cavernous sinus was done until the nerve passed the superior orbital fissure. The long storage time of the samples examined did not allow further histological analysis.

## 3 Results

We focused our investigations on the number of nerve fibers, a comparison between left and right side and macroscopic variations of the AN in the petroclival region, where the AN pierces the dura and switches from the subarachnoid part to the gulfar or petroclival segment in its further course ( 14 ).

### 3.1 Light microscopic observations

We analyzed the number of fibers per nerve in the petroclival region to see if there are differences in number of nerve fibers between the left and right side, taking into account the role of sex and age.

Most of the cross-sections appeared to be oval or irregular shaped with small bundles held together by thin connective tissue layers. The nerve fibers appeared as empty spaces bordered by a ring representing the myelin sheath.

#### 3.1.1 Quantitative observations

The details of the cross-section area of the nerves, as well as general data about our donors, are described in Table 1.

**Table 1:** Number of AN fibres (mean value of three regions and standard deviation) on the right and left side. The superior and inferior bundle of the duplication were handled as one nerve in our statistical analysis.

Case no.	Sex	Age	Right AN, cross sectional area (mm^2^)		Right AN, number of nerve fibres per mm^2^ (estimated number of nerve fibres/cross sectional area)		Right AN, estimated number of nerve fibres		Left AN, cross sectional area (mm^2^)	Left AN, number of nerve fibres per mm^2^ (estimated number of nerve fibres/cross sectional area)	Left AN, estimated number of nerve fibres
1	m	94	1.14		6,002		6,842 (+/−336)		0.83	6,196	5,143 (+/−104)
2	m	89	0.83		4,267		3,520 (+/−713)		1.74	2,599	4,519 (+/−561)
3	f	65	0.43		7,870		3,353 (+/−586)		0.74	7,652	5,678 (+/−463)
4	f	88	0.54		7,220		3,863 (+/−128)		0.32	8,750	2,800 (+/−50)
5	f	84	0.49		10,665		5,194 (+/−224)		0.32	9,428	3,017 (+/−224)
6	f	81	0.82		7,279		5,969 (+/−595)		0.62	8,380	5,171 (+/−95)
7	f	95	0.6		8,728		5,202 (+/−349)		0.57	6,754	3,816 (+/−432)
8	m	83	0.57		9,539		5,390 (+/−617)		0.66	8,355	5,481 (+/−456)
9	f	91	0.69		6,701		4,651 (+/−35)		0.3	9,868	2,990 (+/−223)
10	m	71	0.69		6,908		4,794 (+/−51)		0.66	7,923	5,269 (+/−71)
11	m	82	0.39		10,946		4,258 (+/−78)		0.45	1,161	5,238 (+/−363)
12	f	77	0.66		7,160		4,726 (+/−1,234)		0.92	6,137	5,634 (+/−463)
			Superior bundle: 0.038	Inferior bundle: 0.62	Superior bundle: 4,816	Inferior bundle: 7,327	Superior bundle: 183 (+/−44)	Inferior bundle: 4,543 (+/−514)			

**Figure 1:**
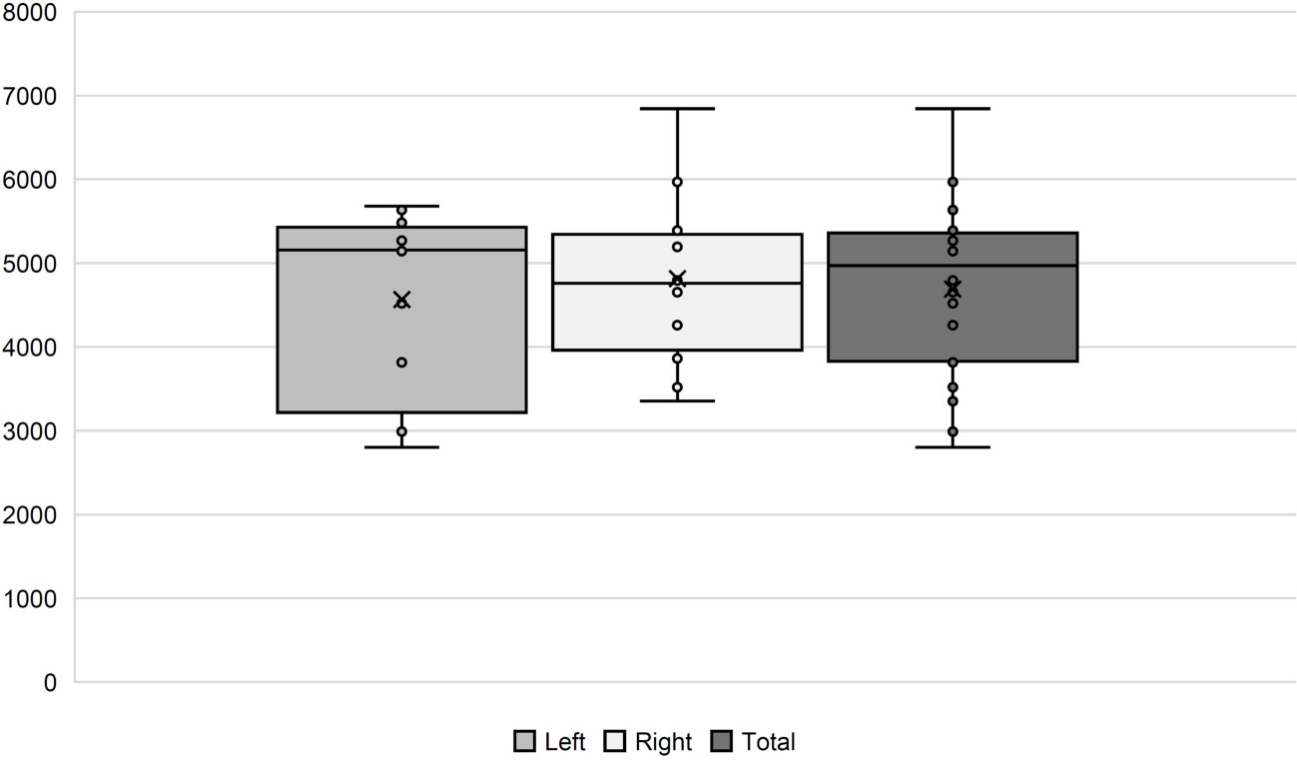
Number of AN fibers in our histological samples. In the boxplots, comparison between the left and right side, as well as the total amount, is shown.

We did count a mean of 4,688 (+/−1,041) nerve fibers per nerve. On average, we found 4,563 (+/−1,547) in the left and 4,814 (+/−1,778) in the right AN. On the left side, the numbers ranged between 2,800 and 5,876, and on the right side, from 3,353 to 6,842 (Figure 1). There is no lateralization in the number of nerve fibers (*p* = 0.27).

The estimated mean of the male donors was 5,045 (+/−877) nerve fibers, while the female donors showed 4,433 (+/−1,103). There was no significant correlation between number of nerve fibers and sex or age.

#### 3.1.2 Histological analysis of the duplication

**Figure 2:**
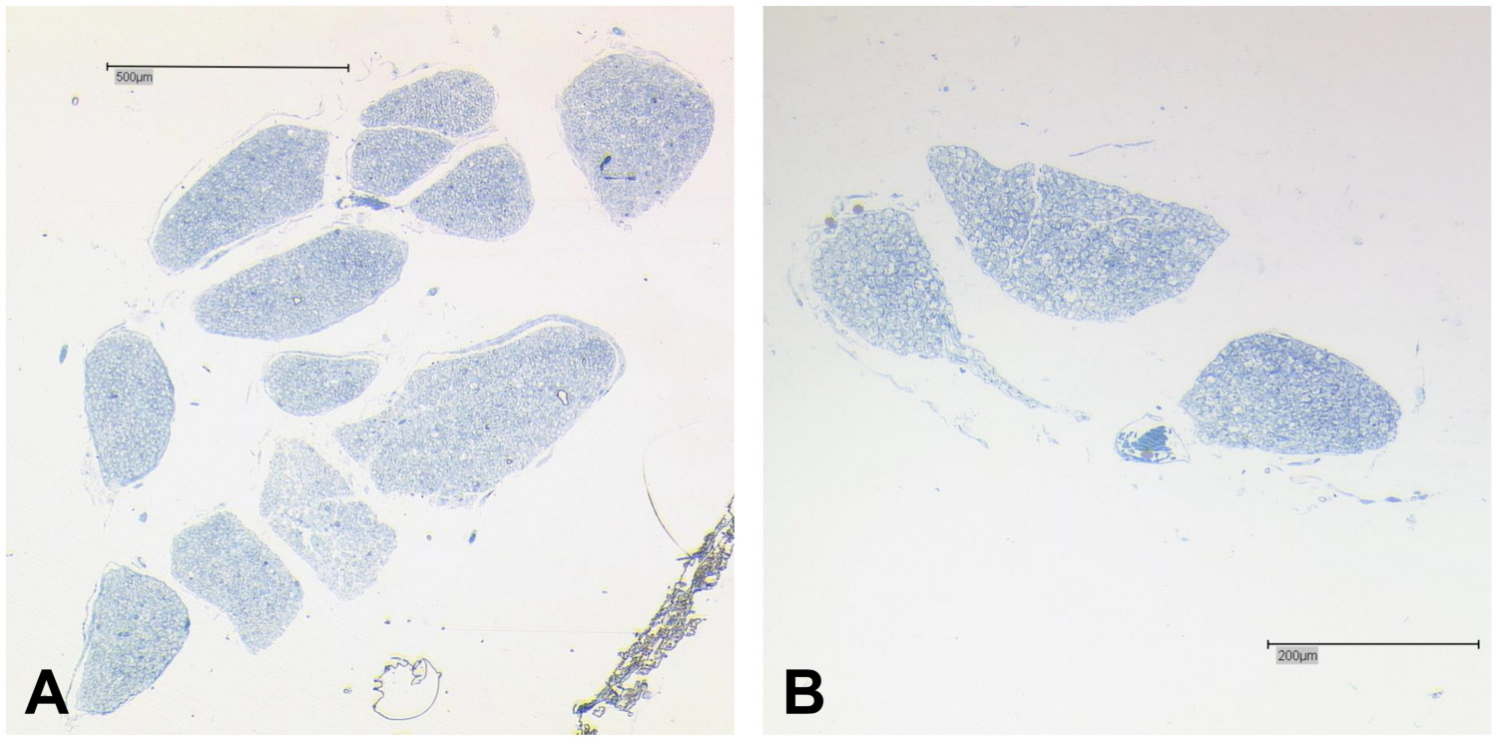
Histology of the duplicated AN. **(A)** Main fiber bundle (inferior bundle) of the AN. The main trunk is organized in twelve bundles that are separated by thin layers of connective tissue. **(B)** Accessory AN branch organized in three small bundles. Bar = 500 μm.

We found a macroscopical duplication of the right AN in a 77-year-old female body donor ( Table 1 : no. 12). We further examined this duplication histologically ( Figure 2 ). There was one main AN branch (inferior bundle) with a cross-sectional area of 0.62 mm^2^ and an accessory branch (superior bundle) with a cross-sectional area of 0.038 mm^2^.

In total, both bundles combined contained an estimated number of 4,726 fibers with the inferior bundle contributing 4,543, and the superior bundle 183 fibers.

### 3.2 Macroscopic examination

**Figure 3:**
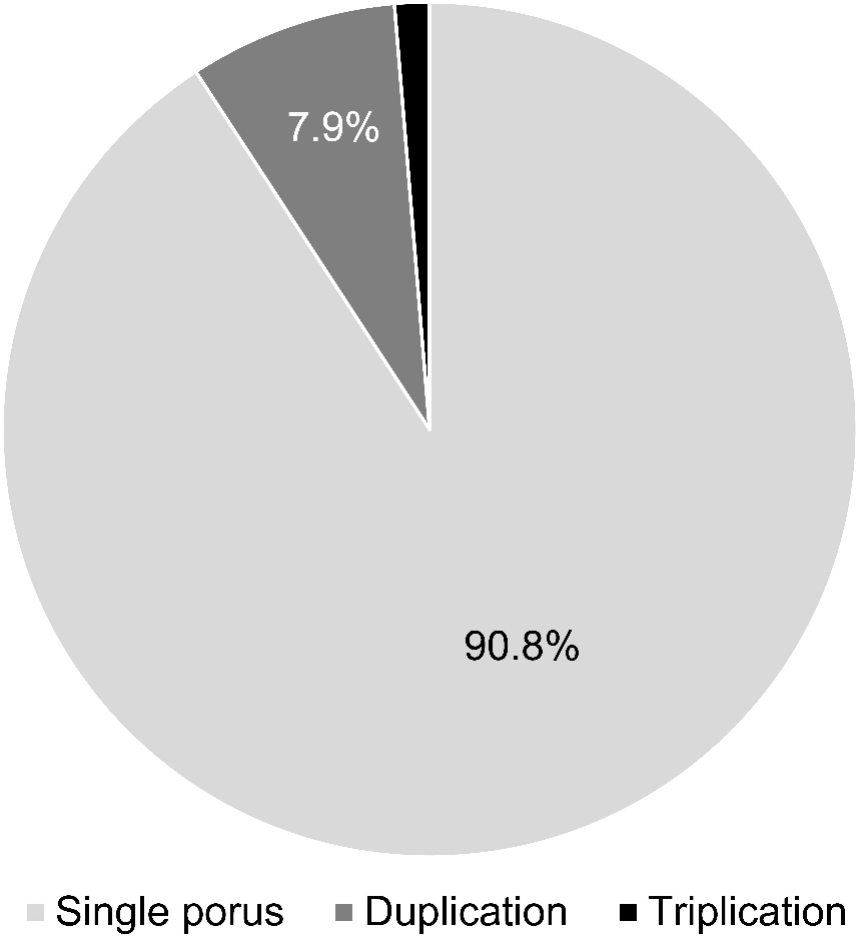
Number of AN dural pori. In most cases (90.8%), only one dural porus was present. Dural porus duplication was observed in 7.9%, and dural porus triplication in 1.3%.

Seven of the 76 skull base sides showed variations in the petroclival region regarding the AN. Figure 3 shows the percentages of duplications and triplications we found.

**Figure 4:**
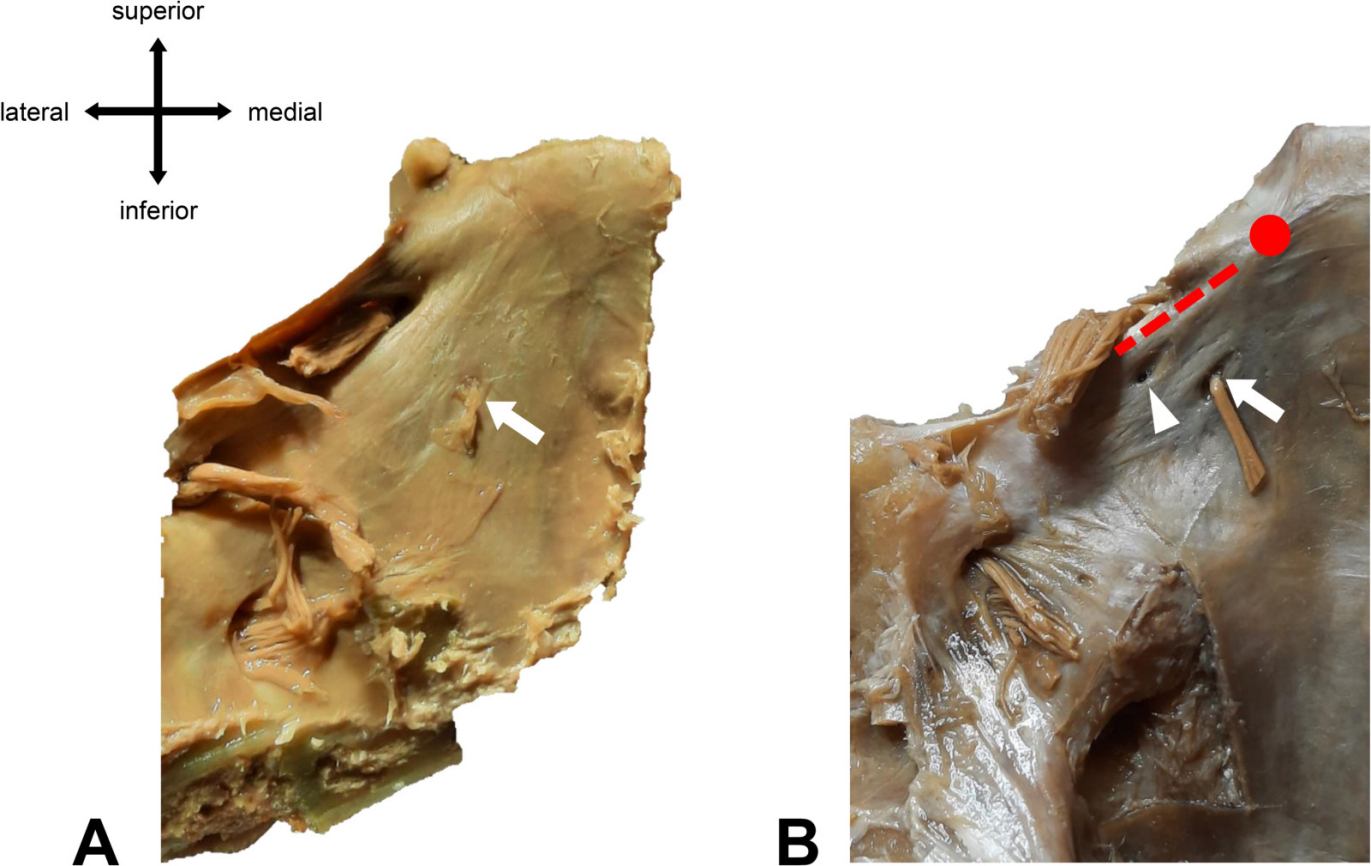
Different types of abducens dural entrances. **(A)** Single left dural porus (white arrow) on the clivus at its textbook position (lateral to the midline of the clivus). **(B)** Duplicated left dural porus. The main dural entrance is marked with a white arrow. The white arrowhead points to the accessory porus, which is located superiorly and laterally to the main porus. Red dotted line = location of Gruber’s ligament. Red dot = location of the posterior clinoid process.

In most cases, only one AN and one single dural porus were observed (90.8%). There were six duplications (four left, two right-sided) (7.9%) and one triplication (right-sided) (1.3%), each with separate dural entrances. The dural pori were, in most cases, slot-shaped, but we also found round pori. Figure 4 shows two examples of our macroscopical findings.

Most of the accessory branches are located superiorly and laterally to the dural entrance of the main trunk. The average distance between the main and the accessory porus was 2.5 mm (+/−1 mm).

### 3.3 Triplication of the AN

**Figure 5:**
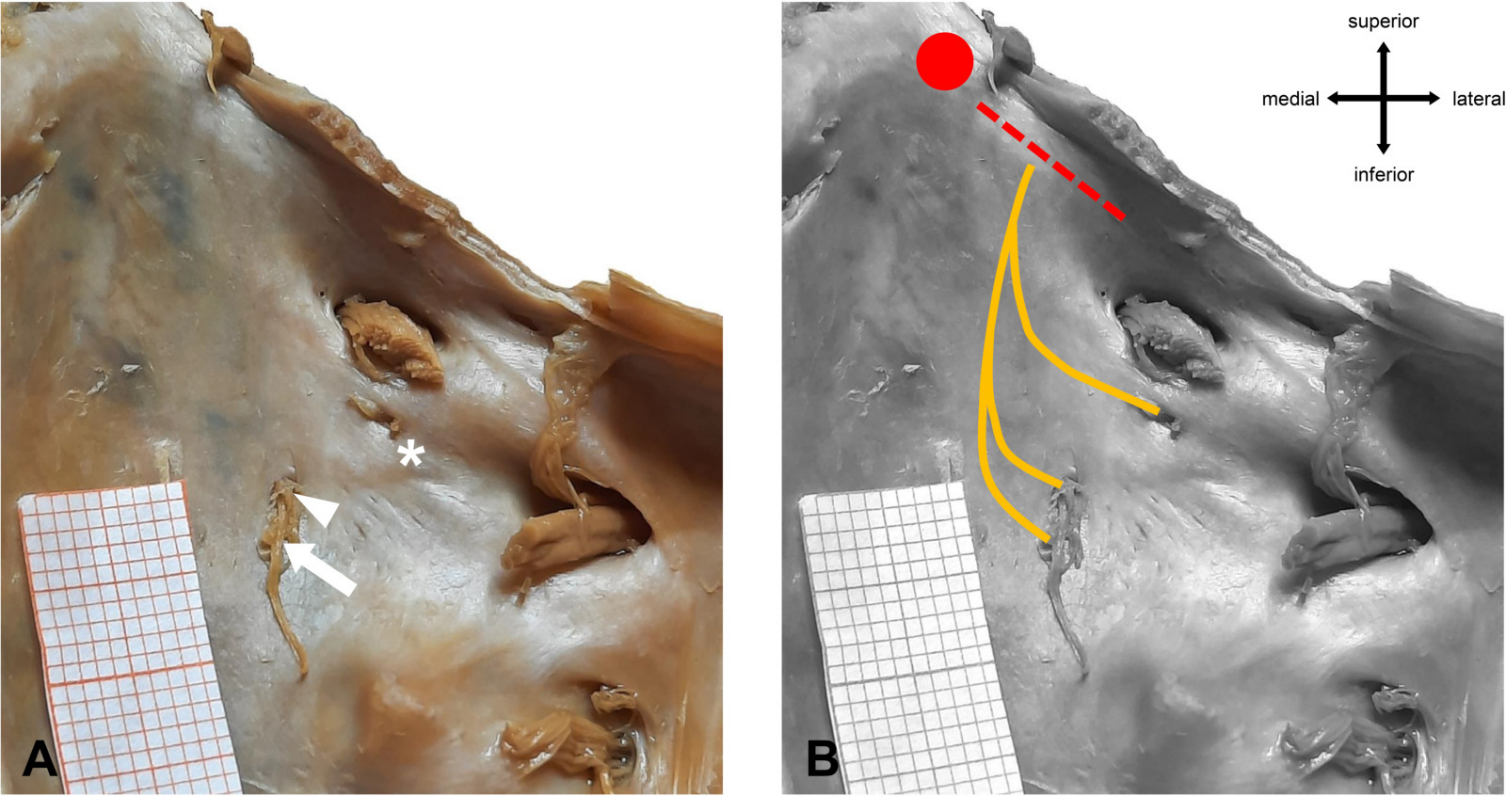
Macroscopic anatomy of AN trifurcation. **(A)** Right side of the clivus with main dural porus (arrow) and two accessory dural pori (arrowhead, asterisk). **(B)** Subdural course of the AN fibers. All three fiber bundles merge before entering Dorello’s canal. Red dotted line = location of Gruber’s ligament. Red dot = location of the posterior clinoid process.

One case of an AN triplication was found at a right skull base side. After carefully removing the dural layer, we could see that all three branches connected closely after piercing the dura mater ( Figure 5 ). The petrosphenoidal ligament (of Gruber) was partly ossified in this case (incomplete Foramen petrosphenoideum osseum anomalum).

## 4 Discussion

The AN in humans has been investigated in multiple previous studies during the last two centuries (24). Here, we discuss our findings focusing on the following topics:

I. Amount of AN fibers
II. L-R-Asymmetry and sex related differences of the AN
III. Macroscopic variations of the AN
IV. Suggestion for a new branching scheme

### 4.1 Different amounts of AN fibers in the literature

We noticed a broad discrepancy in the number of nerve fibers for the AN in literature. The mean estimated number of nerve fibers ranged between 1,997 ( 25 ) and 7,220 ( 26 ) per AN. The first study that investigated this topic in humans was published in 1845 by Rosenthal. He found an estimated mean of 2,000–2,500 fibers per AN in one human. Almost 40 years later, Bors counted 4,698 fibers ( 27 ) in an AN of an adult man.

While several studies in the following years were performed (28, 29), all of them had very limited sample sizes. Interestingly, the numbers of nerve fibers steadily increased. Harley was the first to perform a bigger study regarding the sample size with twenty-four donors (30). Harley investigated the left-right-asymmetry in the amount of nerve fibers and found no significant difference (30). This was confirmed by later studies (31).

Table 2 shows the studies that were previously done on this topic, along with the estimated number of nerve fibers.

**Table 2:** Comparison of different histological studies on the AN. M stands for male; F for female while R stands for right side and L for left side. Data that was not available was indicated by “not given”.

Study	Samples	60 + years	Mean estimated nerve fibers	Sample location	Staining & technique	Comments
Rosenthal (32)	not given	not given	2,000–2,500	not given	Acetum pyrolignosum	
Tergast (33)	not given	not given	3,610	close to the insertion at the LRM	absolute alcohol and carmin staining	
Bors (27)	1 male individual	not given	prox.: 3,862 distal: 4,698	prox.: close to the brainstem distal: close to the insertion at the LRM	not given	Nerve-muscle ratio: 1:5.8
Chiba (34)	3 individuals	not given	5,300	not given	Weigert-Kultschitzky staining	Comparison left/right side
Maleci (29)	2 male individuals	not given	prox.: 6,643 distal: 6,775	not given	25 µm	
Björkman and Wohlfahrt (28)	female individual, 48 years old	0	6,600	close to the brainstem	Glial staining method of Alzheimer-Mann	
Harley (30)	24 indindivi- duals	not given	R: 5,070 (+/−942) L: 5,281 (+/−1,155)	1 cm distal from the brain stem	Bodian silver technique, 10 µm	Comparison left/right side
Swensson (26)	male individual (50 y.o.) and female fetus	0	prox.: 5,310 and 5,890 distal: 5,700 and 7,220	prox.: close to brainstem distal: close to the insertion at the LRM	Glial staining method of Alzheimer-Mann	
Thorsteinsdottir (31)	20 individuals (L 9, R 5)	not given	prox.: R: 3,535 (+/−2,608) L: 3,961 (+/−1,199) distal: R: 3,567 (+/−1,816) L: 3,982 (+/−1,306)	2 mm distal of brainstem and in the cavernous sinus	methylene-blue, 2 µm	Comparison left/right side
Bardosi et al. (35)	10 individuals	not given	1,200 (+/−200)	close to the insertion at the LRM	Nissl staining, 10 µm	Compared the fiber composition in humans and rats
Sawabe et al. (25)	4 male and 6 female individuals, 63–88 years	10	1,997 (+/−432)	sub-arachnoid space	luxol fast blue periodic acid schiff hematoxylin triple stain, 20 µm	Investigated age related changes of the AN
Ramkumar et al. (88)	20 individuals, 12–90 years	5	For age 60+: 4,580 (+/−1,846)	sub-arachnoid space	Toluidine blue stain, 1 µm	Investigated age related changes of the AN
Moriyama et al. (36)	10 male, 10 female individuals	18	F: 1,861 (+/−360) M: 2,110 (+/−506)	not given	luxol fast blue periodic acid schiff hematoxylin triple stain, 15 µm	Comparison sex differences
Present study	5 male, 7 female individuals, 65–94 years	24	R: 4,814 (+/−1,006) L: 4,563 (+/−1,105) F: 4,708 (+/−878) M:−4,961 (+/−1,257)	Petroclival region, right before the dural entrance	methylene blue, 1 µm	Comparison left/right side and sex differences in the same individuals

#### 4.1.1 Impact of sample location

Generally, nerve fibers increase from proximal to distal sample locations. Bors counted 3,862 in the proximal part of the AN and 4,698 in a more distal location (27). While his study only included one male donor of unknown age and ethnicity, this trend was confirmed by other studies with larger sample sizes and also for both sides of the individual (30). It is generally known that the myelinated nerve fibers branch into multiple fibers when traveling from the brainstem to the orbit (26). Sunderland and Hughes added that with its course through the cavernous sinus, the AN is joined mainly by sympathetic bundles of the internal carotid plexus (37). This phenomenon was confirmed with the use of Sihler's stain in a study done by Wysiadecki et al. (7). Some authors suggest that the sample location can be too close to the brainstem; in this case the amount of myelinated nerve fibers is underestimated (26).

#### 4.1.2 Impact of staining technique and slice thickness

Thorsteinsdottir (31) explained the variability of the amount of nerve fibers in literature with different staining techniques. She used methylene blue staining in her work and argued that she might not detect nerve fibers under 2–3 µm in diameter with her methods. According to Tomasch and Schwarzacher (38) most fibers fall in the 2–3 µm category. Furthermore, Agdhur (39) stated that low magnification combined with intense staining could lead to a too-low calculation of nerve fibers and that the differentiation between small nerve fibers and glial cells in the central nervous system is complicated, which could have an influence on why the number counted in samples taken close to the brain stem might be hard to assess.

#### 4.1.3 Impact of ethnicity

Studies of Japanese origin reveal a significantly lower number of AN fibers compared to other populations. Sawabe et al. (25) performed a study with Japanese body donors and reported a mean of 1,997 nerve fibers (10 donors). Moriyama et al. **(**36) found similar numbers in the Japanese population (for females: 1,861 +/−360, for males: 2,110 +/−506). They used samples from 10 male and 10 female body donors with an age of 54–90 years.

### 4.2 Left-right-asymmetry of the AN

In this study we did not find a L-R-Asymmetry of the number of AN fibers.

Studies of asymmetries in other cranial nerves have been published in the past. The morphometry of the recurrent laryngeal nerve differs in humans and rats, with the right nerve generally having thicker fibers (40). Nevertheless, other studies on human cranial nerves showed that in most cases there is no significant lateralization (41). Regarding the abducens nerve, two previous studies compared numbers of left and right nerve fibers (30, 31). Both could not find significant differences in fiber number, but according to Thorsteinsdottir (31), the left AN has more fibers with a diameter of >5 µm measured in the prepontine cistern and the cavernous sinus.

#### 4.2.1 Sex-related differences in AN nerve fibers

We could not show sex-related differences in the number of AN fibers. Moriyama et al. (36) investigated the association between sex and the number of nerve fibers in the AN. They found that there is no sex-related difference in the AN but only used the right AN of the donors (36). In contrast, the vestibulocochlear nerve is sexually dimorphic: female body donors tend to have fewer nerve fibers than male donors (36, 42).

### 4.3 Macroscopic variations of the AN

The following section provides an overview of established theories, as well as recent findings about the branching of the AN.

#### 4.3.1 Literature overview

The prevalence of AN supernumerary variations ranges from 0% to 80% (43, 44) (Table 3).

**Table 3:** Comparison of studies on macroscopic AN variations regarding duplications and triplications.

Authors	Samples	Variations			Comments
		Single trunk	Duplication	Tripl. or more	
Jain (45)	300 cases	94%	6%	–	
Nathan et al. (46)	62 cases	86.5%	11.9%	1.6%	Defined patterns 1–4
Harris and Rhoton (47)	25 cases (50 sides)	68%	26%	16%	Described branching in the cavernous sinus
Piffer and Zorzetto (44)	60 sides	100%	–	–	
DiDio et al. (48)	72 cases	87.5%	11.1%	1.4%	
Umansky et al. (89)	20 sides	85%	15%	–	
Umansky et al. (49)	40 sides	40%	57.5%	2.5%	Investigated the intracranial course
Tekdemir et al. (50)	54 cases	98.2%	1.8%	–	
Destrieux et al. (13)	28 sides	93%	7%	–	Investigated the dural relationships, defined the “petroclival venous confluence”
Ozveren et al. (16)	100 sides	85%	15%	–	Added patterns 5 & 6 to Nathans scheme
Alkan et al. (51)	540 sides	74.8%	25.2%	–	MRI study
Iaconetta et al. (14)	100 sides	92%	8%	–	Defined five anatomical segments of the AN
Romero et al. (52)	17 cases	70%	25%	5%	Described branching exclusively in the cavernous segment
Ozer et al. (53)	40 sides	45%	37.5%	17.5%	Defined “pseudobranching” in the cavernous sinus
Tubbs et al. (19)	24 sides	100%	–	–	Found a tight fixation of the AN at the Dorello canal (“tube within a tube”)
Zhang et al. (54)	104 cases	84%	15%	1%	Defined pattern 7 & 8
Wysiadecki et al. (55)	40 sides	70%	30%	–	
Ipsalali et al. (56)	30 sides	77%	23%	–	Described different patterns regarding the dural entrances of the AN
Iwanaga et al. (90)	36 sides	97.3%	2.7%	–	Described variations of the petroclival ligament
Haladaj et al. (57)	80 sides	96.25%	3.75%	–	Investigated the orbital segments
Kuruc et al. (43)	120 sides	20%	41.66%	23.33%	Investigated branching in the subarachnoid segment
Wysiadecki et al. (7)	60 sides	63.3%	11.7%	–	Described Variants of the cavernous sinus
Rothenstreich et al. (58)	45 sides	82.5%	17.5% pseudo- branching, percentage of duplication not given	–	Investigated comparative anatomy of the abducens nerve at the skull base
Present study	76 sides	90.8%	8%	1.3%	

Other literature reviews found the percentage of duplication for the AN around 7.6% (24) and 5%–28.6% (59). Kuruc et al. (43) described the prevalence of AN variations in the prepontine cistern to be up to 80% in an Eastern European population, which is much higher than any study published before (43).

#### 4.3.2 Embryological development of the AN and variations in development

The somatomotor AN develops early in the hindbrain (rhombomeres r5 and r6). Transcription factors of the myogenic progenitor cells stimulate the extension of the axons out of their respective columns (60). Gilbert stated that the target of the AN, the lateral rectus muscle, originates from two myotomes (61). Different abducens nerve strands could be a result of multiple AN branches that did not fuse together correctly. This theory was brought up first by Bremer, who stated that supernumerary branches of the AN could be the result of failure to resorb aberrant nerve fibers during development (62). Wahl et al. (63) postulated that the innervation of different somitomeres in chick embryos is facilitated by multiple AN branches.

Sato et al. described a change in the AN course during development and proposed that many anatomical structures can alter or modify the course of the AN at various time points of development (64). The developing veins in the parasellar region, around the hypophyseal gland, can alter the course of the AN through the cavernous sinus and separate the oculomotor nerve from the AN. It is shown, however, that the AN has close relations to the ophthalmic nerve, the first branch of the trigeminal nerve. These two nerves are usually separated by an arterial branch of the inferolateral trunk of the internal carotid artery (64). Furthermore, there are case reports about a persistent trigeminal artery (PTA) (65). This artery usually regresses after the manifestation of the posterior communicating artery. In those individuals where it persists or even during development, the PTA has a close relation to the dural entrance of the AN at the clivus and might be able to alter the anatomy of the AN (65). These relations were already described in previous studies as possible cause for a loop-alteration of the AN course (66, 67).

#### 4.3.3 The anterior inferior cerebellar artery

The AN has a close relation to the anterior inferior cerebellar artery (AICA). AN palsy might be associated with compression by the AICA (68) but the close relation could also explain branching of the AN. Descriptions of two abducens roots separated by the AICA which was running between them (14, 45, 49, 69) are found in literature. In a case report, Borg et al. described a 76-year-old woman with an aberrant course of the AN due to compression by an ectatic AICA (70). Additionally, a penetration of the AN by the AICA has been reported (67). In most cases the AICA passes under the AN, so called basal course (67, 71).

#### 4.3.4 Interactions in the cavernous sinus

Sunderland and Hughes (37) found that the AN, after entering the cavernous sinus, splits into multiple bundles, which are separated “through thick dural septa from the fibrous dural prolongation that accompany the AN in its course through the sinus.” Other studies showed similar arrangements with the AN branching in the cavernous sinus close to the posterior aspect of the ICA (10, 47). Ozer first introduced the term pseudobranching for this phenomenon (53). The prevalance of pseudobranching ranges from 17.5% to 37.5% (55, 58). Pseudobranching was also analyzed histologically (7).

#### 4.3.5 The lateral rectus muscle (LRM)

Haladaj found that the lateral rectus muscle has a “dual-headed origin” (72). Nam showed that in most cases a single trunk of the AN branches into two nerves right before the LRM (73). Peng et al. studied the LRM's innervation in primates and humans and could show that the LRM in both species has a compartmentalized innervation with a superior and an inferior zone (74). Branching of the AN might be related to functional units of the RLM (48).

#### 4.3.6 External oculomotor muscles in comparative anatomy

The function of the retractor bulbi muscle (RBM) is to pull the eye further back into the orbit, in response to corneal/trigeminal stimulation. The RBM and the LRM originate from the same blastema. In birds this muscle is divided into two separate portions, the quadratus and pyramidalis muscles (75). Lateral parts of the RBM are innervated by the AN. In most species there are two different cell populations of the AN nucleus found: the dorsal (main or principal) nucleus and the ventral (accessory) nucleus. In frogs (Rana ridibunda) AN fibers that innervate this muscle originate from both the main and accessory abducens nucleus (76). In cats, the sub-nucleus of the AN nucleus innervates the RBM (77). In monkeys the equivalent of the RBM is called the accessory lateral rectus muscle (ALR). Spencer and Porter (78) could show that in some cases both the LRM and the ALR are innervated by the principal abducens nucleus. The ventral abducens nucleus has closer relations to the sensory neurons with trigeminal input, in correlation with the RBM-reflex. Even though this sub-nucleus is not found in humans, these conditions may explain the possibility of two roots of the AN.

### 4.4 Relations to and differentiation from the trigeminal nerve (TN)

Ogut et al. performed a study on 19 formalin-fixed heads and found different shapes of the TN porus (elliptical, oval, and slit-like). They found no variations in the subarachnoid portion of the TN. The estimated distance between the AN and the trigeminal dural entrance was found to be 5.7–9.03 mm for the right and 4.64–8.81 mm for the left side ( 79 ). Another study in the petroclival region also showed no branching variations of the TN ( 80 ). Arslan stated that the mean estimate distance between the trigeminal ganglion and the AN is 1.87 mm ( 81 ).

In a case report, Dupont et al. showed a duplicated AN with a dural porus situated at a similar position like the topmost dural porus in our triplication (82). In this particular case, the ipsilateral roof of the trigeminal porus was ossified. This ossification may be interpreted as a trigeminal bridge, as it has been described by Wegner (83) and Lang (84).

### 4.5 Triplications of the AN are a rare phenomenon

**Figure 6:**
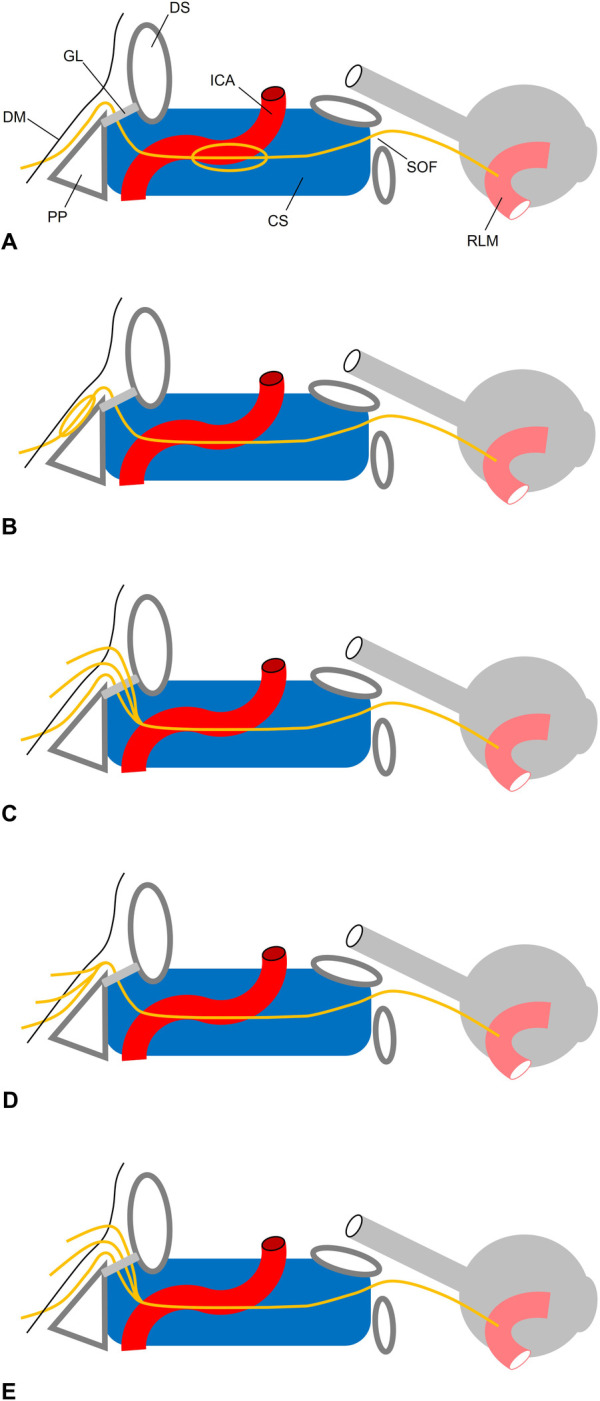
Topography of AN triplication. **(A)** Pseudobranching in the cavernous sinus (CS). **(B)** Pseudobranching under the clival dura mater (DM) as described by (48). The AN pierces the DM through a single porus **(C)** trifurcation as described by (46). Three branches pierce the DM through three dural pori. While the most inferior fiber bunde lies below Gruber’s (GL), the two other bundle lie above the GL. All three bundles merge in the CS. **(D)** Present case. Three branches pierce the DM through three dural pori. All three fiber bundles merge before entering Dorello’s canal. **(E)** Trifurcation as described by (54). Three branches pierce the DM through three dural pori. All fibers lie below GL and merge within the CS at the posterior aspect of the internal carotid artery (ACI). PP, petrous pyramid; DS, dorsum sellae; SOF, superior orbital fissure; RLM, rectus lateral muscle.

DiDio et al. described a triplication in a male (48). In this case, the AN pierced the dura as a single strand, then three nerve branches emerged in the petroclival segment and rejoined before entering the cavernous sinus. Nathan et al. published a case with an AN branching into three trunks in the subarachnoid region, piercing the dura at the clivus separately before rejoining in the cavernous sinus (46). A similar branching was observed by Zhang et al. (54), but all nerve strands passed below the petrosphenoid ligament of Gruber. In our case, the three branches emerging in the subarachnoid space were piercing the dura separately, rejoined to one single strand before passing Dorello's canal. Kuruc et al. described intradural triplications in the prepontine cistern (subarachnoid segment) without investigating their further course (43). Branching in the cavernous sinus are a form of the aforementioned pseudobranching but can also be seen as triplications. Figure 6 illustrates the different types of triplications.

### 4.6 Suggestion for a new classification system

In literature there are various approaches to categorize variations of the AN. The most prominent ones are the morphological categorization by Nathan (46) and later Ovzeren (16) in six distinct branching patterns, and the approach of branching in specific segments by Wysiadecki (55) and Ozer (53). Nathan et al. published the first branching scheme in 1976. They described four different patterns of the AN (Pattern 1). Ozveren et al. added Patterns 5 and 6 to this scheme. Those patterns are based primarily on cases by Tillack and Winer (85) (Pattern 5), Nathan et al. (46) and Jain (45). Zhang added patterns VII (duplication with piercing the dura separately with multiple branching in the cavernous sinus and merging as one trunk before entering the orbit) and VIII (duplication as a loop exclusively in the cavernous segment). Wysiadecki et al. published another scheme regarding the cavernous segment: Type I is a single nerve strand. Type II and III are both duplications of the AN, with type II being reserved for duplications only in the cavernous part of the nerve's course (55).

Iaconetta et al. proposed a five-segment classification for the AN: cisternal, gulfar, cavernous, fissural and intraconal (14). Other authors specified the petroclival region as the AN segment starting at the dural entrance and ending in the Dorello's canal (53, 56). Studies show that this region is of considerable interest when investigating variations (53). More recently, Ipsilali published a variation scheme that investigated the petroclival region (Ipsilali et al., 2019) and focused on the dural entry pores but did not further investigate the AN course.

**Figure 7:**
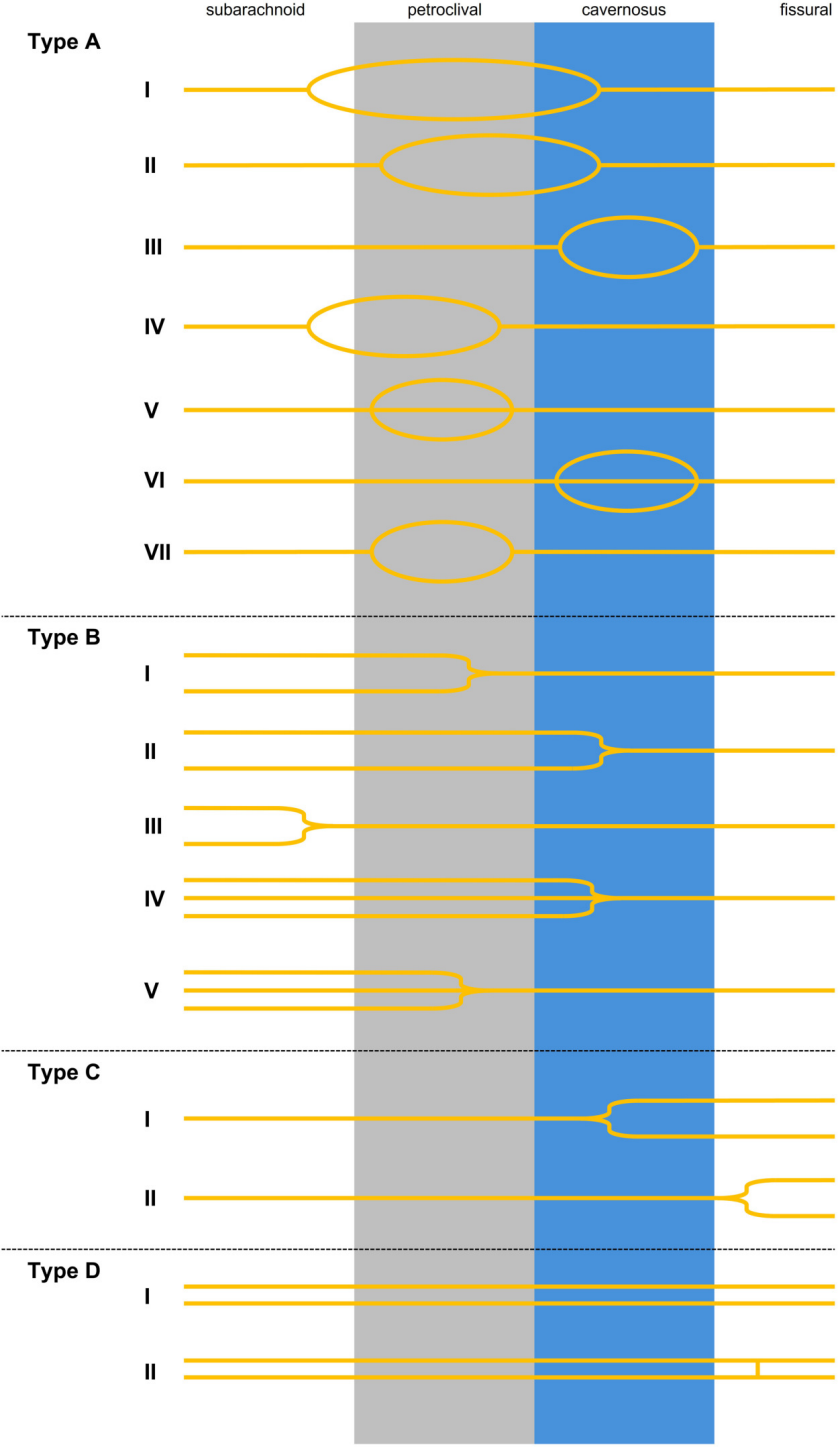
Categories of the AN branching: A = loop shaped AN. AI: loop starts in the subarachnoid segment and the branches merge in the cavernous sinus (12, 46, 50, 54, 55, 69, 91). AII: looping starts in the petroclival segment and merging in the cavernous sinus (92). AIII: looping exclusively in the cavernous sinus (17, 49, 53–55). AIV: looping starts in the subarachnoid region and merging in the petroclival segment (55). AV: Tripliation exclusively in the petroclival region DiDio et al. (48). AVI: pseudobranching into three or more rootlets exclusively in the cavernous segment (17, 47, 52, 53, 55). AVII: looping exclusively in the petroclival region (49). Type B: multiple trunks merging. BI: two trunks merging in the petroclival segment (49). BII: two trunks merging in the cavernous sinus (7, 12, 45, 46, 49, 53–55, 69), BIII: two trunks merging in the prepontine cistern (49, 54). BIV: three trunks merging in the cavernous sinus (46, 54). BV: three trunks merging in the petroclival segment (present study). C = one strand splitting into multiple trunks. CI: one strand splitting into two trunks in the cavernous sinus (7, 53). CII: one strand splitting into two trunks in the fissural segment (57). D = two separate trunks travelling from the brainstem to the orbit. DI: two nerve strands travelling from brainstem to orbit (45, 86). DII: Variation in which the two branches travel separately and have a short connection at the fissural segment (87).

To describe the branching in a systematic way we suggest using four different main categories A-D (Figure 7). Each category stands for a different branching pattern: Category A is a loop shaped branching, starting as a single trunk branching into two or more roots before merging to one trunk again. This variation can be mostly seen in the petroclival segment of the AN (12, 46, 48–50, 53–55, 69). Category B are two or more roots exiting the brainstem merging to one nerve strand during their course at the skull base. Most of the merging is observed at the petroclival segment and at the start of the cavernous segment (49). Category C is a Y-shaped pattern with the AN starting as a single trunk branching into two or more roots innervating the LRM separately. These variations are observed in the orbital segments and the cavernous segments and can be seen as early branching (7, 53, 57). Category D shows reports of two trunks of the AN travelling from the brain stem to the orbit separately. This variation was only described by Jain and Testut (45, 86). Wysiadecki et al. published a somewhat similar case with the difference of the two trunks merging in the orbit before rebranching again (87).

Our branching scheme shows that the most variability of the AN is displayed in the petroclival region, mostly in the morphological form of a loop shape (Category A) or multiple roots merging to a single trunk (Category B).

### 4.7 Limitations of this study

As our study was limited to already formalin-fixated body donors who underwent the fixation process in some cases multiple years ago, histological analysis was not possible in all cases. Especially, we were not able to perform immunohistochemical analysis on our material.

### 4.8 Future investigations

In this study, we focused on the petroclival region, because of its importance in skull base surgery. In the future, it could be interesting to study the AN and its variations in other anatomical areas. Furthermore, it would be advantageous to analyze the branching of the AN with immunohistochemical methods that allow to differentiate fiber qualities. Additionally, systematic analysis of different populations would be advantageous.

## 5 Conclusion

This study aims to provide recent morphometric data for the abducens nerve in body donors of the University of Mainz, Germany. With over 8% of macroscopic variations in the petroclival region, our results might be relevant for neurosurgery in this location.

## Data Availability

The original contributions presented in the study are included in the article/Supplementary Material, further inquiries can be directed to the corresponding author.
